# Dr. Fred L. June

**Published:** 1911-07

**Authors:** 


Dr. Fred L. June, died at his home in Waterport, N. Y., June 6, 1911, aged 56 years. Dr. June was graduated from the medical department of the University of Buffalo in the class of 1876. He is survived by his wife and two daughters, Mrs. Thomas Hartley, of Seattle, Wash., and Miss Bertha L. June. For the past 12 years Dr. June was postmaster of Waterport and for a number of years was coroner of the Lakeside district.

